# Stress-impaired reward pathway promotes distinct feeding behavior patterns

**DOI:** 10.3389/fnins.2024.1349366

**Published:** 2024-05-09

**Authors:** Yusuke Fujioka, Kaori Kawai, Kuniyuki Endo, Minaka Ishibashi, Nobuyuki Iwade, Dilina Tuerde, Kozo Kaibuchi, Takayuki Yamashita, Akihiro Yamanaka, Masahisa Katsuno, Hirohisa Watanabe, Gen Sobue, Shinsuke Ishigaki

**Affiliations:** ^1^Molecular Neuroscience Research Center, Shiga University of Medical Science, Otsu, Japan; ^2^Department of Neurology, Nagoya University Graduate School of Medicine, Nagoya, Japan; ^3^Research Division of Dementia and Neurodegenerative Disease, Nagoya University Graduate School of Medicine, Nagoya, Japan; ^4^Institute for Glyco-core Research (iGCORE), Nagoya University, Nagoya, Japan; ^5^Research Project for Neural and Tumor Signaling, Institute for Comprehensive Medical Science, Fujita Health University, Toyoake, Japan; ^6^Department of Physiology, School of Medicine, Fujita Health University, Toyoake, Japan; ^7^Chinese Institute for Brain Research, Beijing (CIBR), Beijing, China; ^8^Department of Neurology, School of Medicine, Fujita Health University, Toyoake, Japan; ^9^Aichi Medical University, Nagakute, Japan

**Keywords:** feeding behavior patterns, fixated feeding, psychosocial stress, biomarker, dopamine, reward system

## Abstract

Although dietary behaviors are affected by neuropsychiatric disorders, various environmental conditions can have strong effects as well. We found that mice under multiple stresses, including social isolation, intermittent high-fat diet, and physical restraint, developed feeding behavior patterns characterized by a deviated bait approach (fixated feeding). All the tested stressors affected dopamine release at the nucleus accumbens (NAcc) shell and dopamine normalization reversed the feeding defects. Moreover, inhibition of dopaminergic activity in the ventral tegmental area that projects into the NAcc shell caused similar feeding pattern aberrations. Given that the deviations were not consistently accompanied by changes in the amount consumed or metabolic factors, the alterations in feeding behaviors likely reflect perturbations to a critical stress-associated pathway in the mesolimbic dopamine system. Thus, deviations in feeding behavior patterns that reflect reward system abnormalities can be sensitive biomarkers of psychosocial and physical stress.

## 1 Introduction

Feeding behaviors in humans and other animals are tightly regulated by diverse regulatory brain circuits, primarily involving the hypothalamus and its neural circuits, integrating signals from the body and the brain. Other brain regions, including the brainstem and reward centers like the mesolimbic dopamine system, also play significant roles in modulating feeding behavior based on food’s reward value (Saper et al., 2002; Bouret et al., 2004; Morton et al., 2014; Rossi and Stuber, 2018). Quantitative aspects of feeding behaviors are defined by the degree of food consumed and are mainly controlled by hypothalamic circuits and peripheral metabolic hormones in response to energy demands (Berthoud and Morrison, 2008; Sasaki, 2017). In contrast, qualitative aspects depend on the identification of feeding patterns, the underlying biological processes of which remain to be fully clarified. Therefore, eating disorders have been typically diagnosed on food consumption instead of the patterns (Erzegovesi and Bellodi, 2016).

Feeding behaviors can be affected by various stressors (Rojo et al., 2006; Adam and Epel, 2007; Bemanian et al., 2020) as well as neuropsychiatric disorders such as autism spectrum disorders (ASD) and frontotemporal lobar degeneration (FTLD) (Ikeda et al., 2002; Ahmed et al., 2016; Bandini et al., 2017). Recent studies have also found that food addiction is notably more prevalent in individuals with Parkinson’s disease, independent of medication type or dosage, indicating potential inherent abnormalities in eating behavior patterns (Solinas et al., 2019; de Chazeron et al., 2021). Moreover, deviations in food preferences, attributed to altered reward processing in both animals and humans (Adam and Epel, 2007; Alonso-Alonso et al., 2015), highlight that qualitative changes in feeding behaviors could act as indicators for impaired reward systems associated with stressors and neuropsychiatric conditions.

Our understanding of the mechanisms underlying how various stress conditions disrupt normal feeding behavior patterns is limited. The necessary insights needed to advance research into these patterns will require the development of quantifiable methods that assess factors other than consumption. This approach will shed light on the disease mechanisms underlying feeding behaviors, offering insights into both the qualitative and quantitative aspects similar to evaluations of sleep disturbances (Toth and Bhargava, 2013; Wesselius et al., 2018).

In the present study, we developed a real-time monitoring system to assess feeding behavior patterns as a means to more accurately detect the physiological state of subjects. Using the system, we found that different stress conditions induced alterations to the feeding patterns. These deviations, occurring independently of metabolic alterations, exhibited a strong correlation with impairments in the mesolimbic dopamine system. Therefore, they could serve as an ideal biomarker for neuropsychiatric disorders characterized by alterations in reward systems.

## 2 Method details

### 2.1 Mice and stress models

To investigate the effects of isolation stress on feeding behaviors, we randomly assigned wild-type C57BL/6J strain mice to either an experimental group that housed solitary mice or a control group. For the control group, mice were housed together in groups of four according to standard animal care protocols. For the experimental group, mice were housed alone for 7 days prior to the behavioral experiments and *in vivo* microdialysis. Social isolation is an environmental stressor that can alter feeding behaviors but does not necessarily affect body weight (Yamada et al., 2015). It is thus a good model for elucidating the mechanisms underlying altered feeding behavior patterns in response to stress that is not accompanied by alterations in food intake and body weight.

Temporally limiting access to palatable diets is a common method to induce binge eating behaviors in rodents (Avena and Bocarsly, 2012). As a stress model for increased food intake and body weight, we established an intermittent high-fat diet (HFD) model by randomly assigning 6-week-old, inbred mice (C57BL/6J strain) to either an intermittent HFD group or a control group. The mice were placed in cages with free access for both standard chow diet and drinking water except for HFD treatment. The intermittent HFD group could access HFD (Test Diet 58Y1, PMI Nutrition International, KS, United States; 23.1% protein, 34.9% fat, 25.9% carbohydrate, and 6.5% fiber) for 2 h every other day, whereas *ad libitum* supply of standard chow (CE2, CLEA, Tokyo, Japan; 24.8% protein, 4.6% fat, 49.9% NEF, and 4.65% fiber) was used for the control group mice. After 2 weeks of HFD treatment, mice from the experimental and control groups were subjected to real-time monitoring of feeding behaviors and *in vivo* microdialysis.

To investigate the effects of physical stress on feeding behaviors that are accompanied by reduced food intake and body weight, inbred C57BL/6J mice were randomly assigned to either a restrained group or an unimpeded movement control group. Seven-week-old mice in the restrained group were completely immobilized using disposable mouse DecapiCone restrainers (MDC-200, Braintree Scientific, Inc. MA, United States) for 2 h over five consecutive days prior to assessing feeding behaviors or *in vivo* microdialysis analyses. Detailed protocols have been described elsewhere (Besnard et al., 2018). Behavioral analyses were initially performed using male mice followed by validation using female mice as indicated in the individual figure legends.

Dopamine transporter (DAT)-Cre mice (B6.SJL-*Slc6a3^tm1.1(cre)Bkmn^*/J) were obtained from Jackson Laboratories. The tyrosine hydroxylase transporter (TH)-Cre mice (B6.FVB(Cg)-Tg(Th-Cre)FI172Gsat/Mmucd) were obtained from MMRRC.

All animal experiments were performed in accordance with the National Institute of Health Guide for the Care and Use of Laboratory Animals and were approved by the Nagoya University Animal Experiment Committee and the Shiga University of Medical Science Animal Care and Use Committee. All methods were carried out in accordance with relevant guidelines and regulations. All methods are reported in accordance with ARRIVE guidelines. Water was provided *ad libitum*.

### 2.2 Real-time monitoring system

To eliminate any potential artificial effects on the reward system, we developed a real-time monitoring system in tandem with regular diet instead of palatable foods and operant conditions to assess feeding behaviors. This approach allows us to visualize subtle alterations in ordinary feeding behaviors of animals fed a regular diet.

The real-time monitoring system consists of a box containing standard chow diet (CE2, CLEA) affixed to the surface of a 60-cm diameter circle originally designed for open field tests. Four bait containers (33 × 37 × 22 mm, SHINFACTORY, Fukuoka, Japan) were affixed at 10-cm intervals along the outer edge of one-half of the circle plate in close proximity to the wall (height, 40 cm). The respective diet containers were designed such that the mice needed to physically insert their heads into the container to consume the bait. This movement was captured and analyzed with the EthoVision XT 12 (Noldus Information Technology, Wageningen, the Netherlands) motion capture imaging system. The software was used to monitor approaches to bait containers and the duration of each feeding movement in the arena. Prior to the start of the experiment, mice were deprived of a food source for 4 h, after which their feeding behavior was observed for 4 h using the real-time monitoring system. At the conclusion of the experiment, the residual bait in each container was weighed.

### 2.3 Definitions of fixated feeding

Using the real-time monitoring system, we defined the distinct aberrant feeding behavior pattern phenotype as “fixated feeding,” which was characterized by an abnormal deviation in food selection. For instance, mice with spatial fixation approached a specific bait container more frequently than the other containers, whereas control mice had no bait preference.

### 2.4 Kinetic measurement of dopamine levels by *in vivo* microdialysis

The amount of dopamine in the NAcc shell was measured by microdialysis. The guide cannula was stereotaxically placed into the right side of the mouse NAcc shell (L: +0.42, A: +1.34, H: −3.95) a week prior to the measurement. Mice were anesthetized via intraperitoneal injection with a mixed anesthetic agent consisting of medetomidine (0.3 mg/kg), midazolam (4 mg/kg), and butorphanol (5 mg/kg). Experimental mice were deprived of food for 15 h prior to the start of the assay. Dopamine levels were monitored by HPLC-ECD (HTEC-510, Eicom, Kyoto, Japan) both before and after feeding. Dialysis probe (FX-6-01, Eicom) placements were verified histologically at the ends of each experiment, and experimental data were excluded if the membrane portions of the dialysis probes lay outside the NAcc shell region.

### 2.5 Selective dopamine administration into the NAcc shell

Mice under the three experimental conditions selectively had dopamine administered into the NAcc shell. The guide cannula was stereotaxically placed into the right side of the mouse NAcc shell (L: +0.42, A: +1.34, H: −4.25) 1 week prior to assessing feeding behavior. Mice were properly anesthetized as described above. The dopamine solution was prepared as 3-hydroxytyramine hydrochloride: C_8_H_11_NO_2_-HCl (Tokyo Chemical Industry, Tokyo, Japan) diluted in solution buffer [0.5% ascorbic acid (ASA; Iwaki Seiyaku, Tokyo, Japan)/Ringer’s solution]. Dopamine administration was performed 5 min prior to feeding behavior analyses by injecting 0.5 μL dopamine solution (200 μg/μl) into the NAcc shell at a flow rate of 0.25 μL/min. The injection cannula was maintained in place post-injection for 3 min. For control mice, the same volume of 0.5% ASA/Ringer’s solution was injected into the NAcc shell.

### 2.6 DREADD system

To investigate the functional properties of the dopaminergic neurons in the ventral tegmental area (VTA), we used a DREADD system employing AAVs expressing hM4Di with subsequent clozapine N-oxide (CNO, R&D Systems, Minneapolis, MN) administration. We injected 0.5 μL of AAV-mCherry-FLEX-hM4Di virus (2.0 × 10^13^ copies/ml) into the bilateral VTA regions (L: +/−0.4, A: −3.28, H: −4.50) of 6-week-old DAT-Cre mice and TH-Cre mice at a flow rate of 0.25 μL/min. The injection cannula was maintained in place post-injection for 3 min. Mice were properly anesthetized as described above. CNO [1.0 μg/g of BW, 0.1 μg/μl in 10% DMSO (Sigma-Aldrich, St. Louis, MO) -saline mixture] was intraperitoneally administered 30 min prior to real-time monitoring of feeding behavior or *in vivo* microdialysis. For controls, the same volume of 10% DMSO-saline mixture was administered intraperitoneally.

### 2.7 Body composition and tissue examination

Body weight, rectal temperature, and blood glucose were monitored in 8-week-old mice. Dissection and collection of tissue samples including brain, spleen, pancreas, white adipose tissue (WAT), and brown adipose tissue (BAT) were likewise performed using 8-week-old mice synchronized to the same time schedule as mice in the microdialysis or feeding behavior monitoring experiments. Mice were properly anesthetized as described above. Mice were allowed *ad libitum* access to water and standard bait prior to dissection. Blood glucose levels were measured before anesthetization. After cooling on ice, the brain was sectioned with a 1-mm thick brain slicer (MBS-A1C, BrainScience idea, Osaka, Japan), and the hypothalamus was collected under a stereomicroscope and rapidly cooled to −80°C along with the WAT and BAT. Rectal temperature measurements and plasma catecholamine concentration determinations were performed immediately after each final stress under anesthesia. Rectal temperatures were measured by inserting a probe (TX10-01, 900-21B, Kenis, Osaka, Japan) coated with mineral oil (Sigma-Aldrich) into the anus and fixing it at a distance of 2-cm, and then measuring the temperature after stabilization. The plasma catecholamine concentration was measured by intracardiac blood sampling with samples immediately placed in EDTA-2 K anticoagulant spits (BD Japan, Tokyo, Japan) and centrifuged at 1,500 × g for 10 min. The supernatant was collected and passed through a clean EG column (CA-50 PBA, Eicom) prior to sequential HPLC-ECD (HTEC-510, Eicom) loading to determine the catecholamine concentration. 3,4-dihydroxybenzylamine (858781, Sigma-Aldrich) was used as an internal standard.

### 2.8 Immunohistochemistry

Mouse brains were fixed in 4% paraformaldehyde and cut into 40-μm-thick sections using a vibratome (Leica Biosystems, Nussloch, Germany). The sections were blocked using blocking solution (X0909, Agilent Technologies, Santa Clara, CA) for 1 h followed by overnight incubation with anti-TH antibody (AB152, Sigma-Aldrich) diluted in antibody diluent (S0809, Agilent Technologies) at 4°C. For immunofluorescence, Alexa-488-conjugated secondary antibody (Thermo Fisher Scientific, Waltham, MA) and DAPI Fluoromount-G (Southern Biotech, Birmingham, AL) were used.

### 2.9 Real-time qPCR for gene expression analysis

An RNeasy Mini Kit (Qiagen, Hilden, Germany) was used to isolate total RNA from tissues. cDNA synthesis was subsequently performed using 1 μg total RNA and oligo-dT primers (C110A, Promega, Madison, WI). Transcript levels were assessed via real-time quantitative polymerase chain reaction (qPCR) amplifications using an iQ SYBR Green Supermix (BioRad, Hercules, CA) with primers listed in Supplementary Table S1 on a CFX96 system (BioRad). Thermocycler conditions were 95°C for 3 min followed by 40 cycles of 95°C for 10 s and 55°C for 30 s. The relative quantity of each transcript was determined via a standard curve using the cycle thresholds of cDNA serial dilutions normalized to *Gapdh* levels. PCR was performed in triplicate for each sample, and all experiments were replicated twice.

### 2.10 Statistical analysis

Statistical analyses were performed using JMP 15 (SAS Institute, Drive Cary, NC). An Anderson-Darling test was utilized to assess normality within each group. In cases where normality was not rejected, Welch’s *t* test was applied for groups without correspondence, whereas a paired *t* test was employed for groups with correspondence. Conversely, when normality was rejected, a Mann–Whitney U test was conducted for groups lacking correspondence, and the Wilcoxon signed-rank test was applied for groups with correspondence. Pearson’s chi-square test was applied to assess the significant association between two categorical variables. These procedures are detailed in the figure legends, with statistical significance established at *p* < 0.05. Significant differences in groups were determined by ANOVA with repeated measures as indicated in the Figure legends of *in vivo* microdialysis. The data are expressed as mean ± SEM. All statistical tests were two-sided.

## 3 Results

### 3.1 Psychosocial and physical stresses cause aberrant feeding behavior patterns characterized by fixated feeding

**Figure 1:**
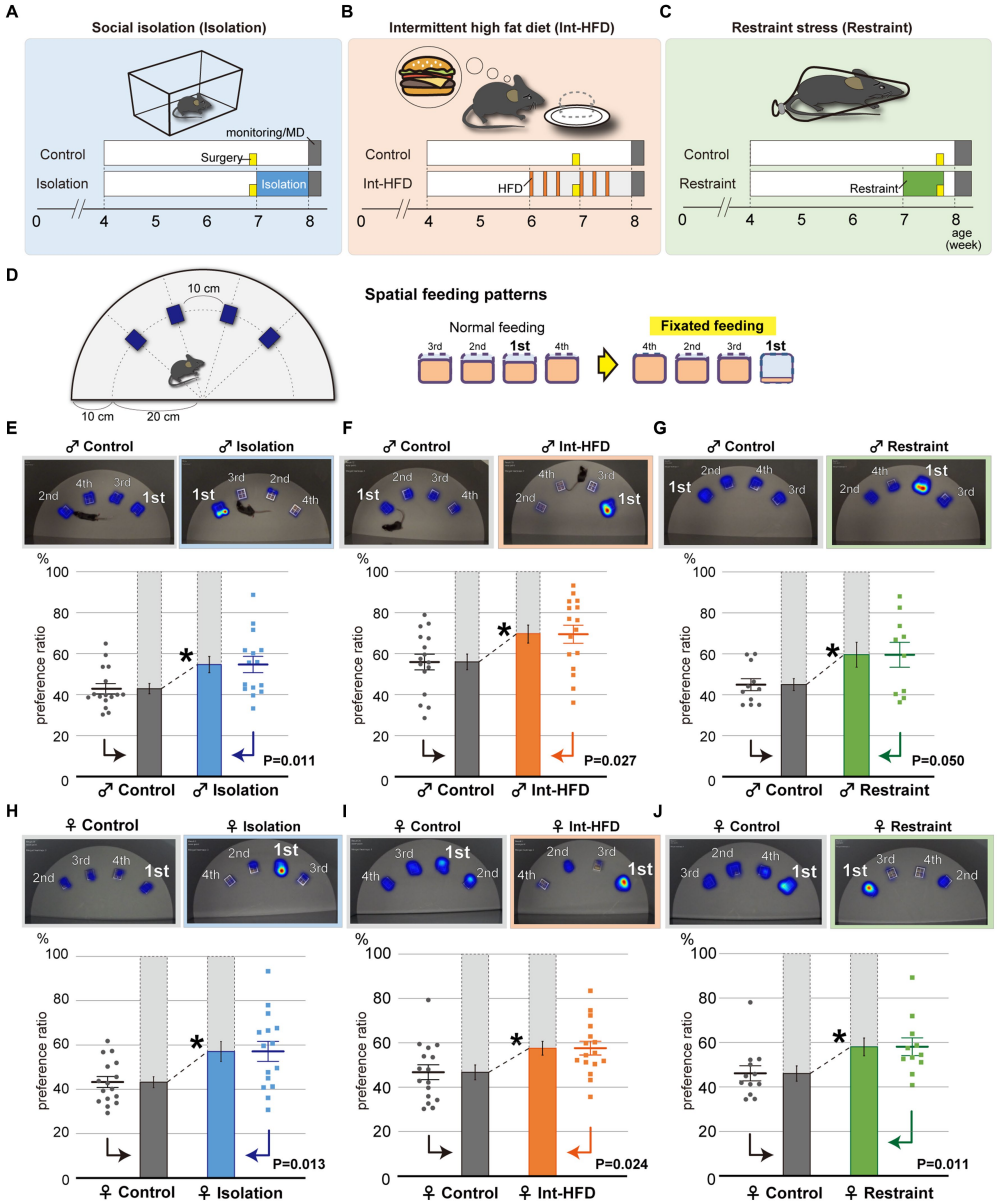
Psychosocial and physical stresses such as social isolation, intermittent high-fat diet, and physical restraint cause aberrant feeding behavior patterns characterized by fixated feeding. **(A–C)** Study paradigms for social isolation (**A**, Isolation), intermittent high-fat diet (**B**, Int-HFD), and restraint stress (**C**, Restraint) are shown. Wild-type C57BL/6 J strain mice were assigned to either stressor or control groups. Mice in social isolation were maintained alone in cages for a week (blue). Mice in the intermittent HFD group accessed a HFD for 2 h during the day every other day for 2 weeks (orange). For the restraint group, 7-week-old mice were subjected to 2 h of complete immobilization using restrainers for five consecutive days (green). Yellow boxes indicate the day when the cannulas were placed into the NAcc of mice for microdialysis experiments. **(D)** Experimental scheme to assess mouse feeding behaviors. Four bait containers were affixed at 10-cm intervals along the outer edge of half a circle plate. The respective diet containers required mice to physically insert their heads to consume the bait. Mouse movements were captured and analyzed using imaging software. Although mice normally feed rather equivalently from multiple bait sources, when mice show a clear preference for a specific food source, the feeding pattern was defined as “fixated feeding.” **(E)** The spatial feeding patterns of male mice reared in social isolation or in group housing (Control). Representative heatmap images indicate the duration of time spent on a bait source. The duration on each bait source was quantified with a motion capture system and the sources ranked in decreasing order of duration at each bait position and a preference ratio was determined. Similarly, the amount consumed from each source was quantified with the sources ranked in decreasing order of bait consumption (Supplementary Figures S1A–C). Statistical analysis was performed on the primary preference ratio (*n* = 15 for Isolation, *n* = 16 for Control, Welch’s *t* test). **(F)** The spatial feeding patterns of male mice with the intermittent HFD diet or a normal diet (Control). Representative heatmap images reflect the duration of time spent on a bait source as before. The duration at each bait source was quantified with a motion capture system and statistical analysis were performed as in **(E)** (*n* = 16 for each, Welch’s *t* test). **(G)** The spatial feeding patterns of physically restrained and non-restrained male mice (Control). Representative heatmap images, quantification of the duration of time spent at each source, and statistical analysis were performed as in **(E)** (*n* = 10 for Restraint, *n* = 11 for Control, Welch’s *t* test). **(H)** The spatial feeding patterns of female mice reared in social isolation or in group housing (Control). Representative heatmap images indicate the duration of time spent at a bait source. The duration spent at each bait source was quantified using the motion capture system with the sources ranked in decreasing order of duration at each bait position and a preference ratio was determined. Statistical analysis was performed on the preference ratio (*n* = 15 for Isolation, *n* = 16 for Control; Welch’s *t* test). **(I)** The spatial feeding patterns of female mice reared on the intermittent HFD or a normal diet (Control). Representative heatmap images reflect the duration of time spent at each bait source as before. The duration of time at each bait source was quantified with the motion capture system (*n* = 16 for both; Welch’s *t* test). **(J)** The spatial feeding patterns of physically restrained and non-restrained female mice (Control). Representative heatmap images, quantification of time spent at each source (*n* = 11 for Restraint, *n* = 12 for Control; Mann–Whitney U test). *p < 0.05. Data are mean ± SEM.

To investigate how feeding behavior patterns are regulated, we used a mouse model incorporating three different stressors: social isolation, a major inducer of anxiety (Figure 1A); intermittent high-fat diet (HFD), which provokes binge-like eating behaviors and is a broad sense stressor (Figure 1B) (Kalyani et al., 2016; Turton et al., 2017); and physical restraint, which mimics the physical stress caused by confinement (Figure 1C). Both stress conditions and neuropsychological disorders can induce aberrant feeding behavior patterns such as fixations on one kind of food or overeating regardless of energy demands. To detect these aberrations, we examined impaired feeding states in terms of spatial feeding behavior patterns defined as “fixated feeding,” a preference for a particular bait. Spatial feeding patterns were assessed using a monitoring system that captured mouse movements during feeding in a field containing four stationary bait containers (Figure 1D).

By calculating the duration of time spent per bait position and the amount of bait consumed, we found that socially isolated mice fixated on a specific bait location. In contrast, feeding in the control group was more evenly distributed across the different bait positions (Figure 1E, Supplementary Figure S1A). Similar fixation on a specific bait location was also observed in mice from the intermittent HFD and physically restrained groups (Figures 1F,G, Supplementary Figures S1B,C), indicating a role for stress in provoking the fixated feeding behavior. No significant preferences for a specific position were observed for any of the groups in their selection of the most preferred bait container (Supplementary Figure S1D). Further, no sex differences were found in the aberrant feeding behavior patterns (Figures 1H–J, Supplementary Figures S1E–G).

### 3.2 Dopamine release in the nucleus accumbens shell is impaired by psychosocial and physical stresses, whereas dopamine supplementation normalizes stress-induced fixated feeding behaviors

**Figure 2:**
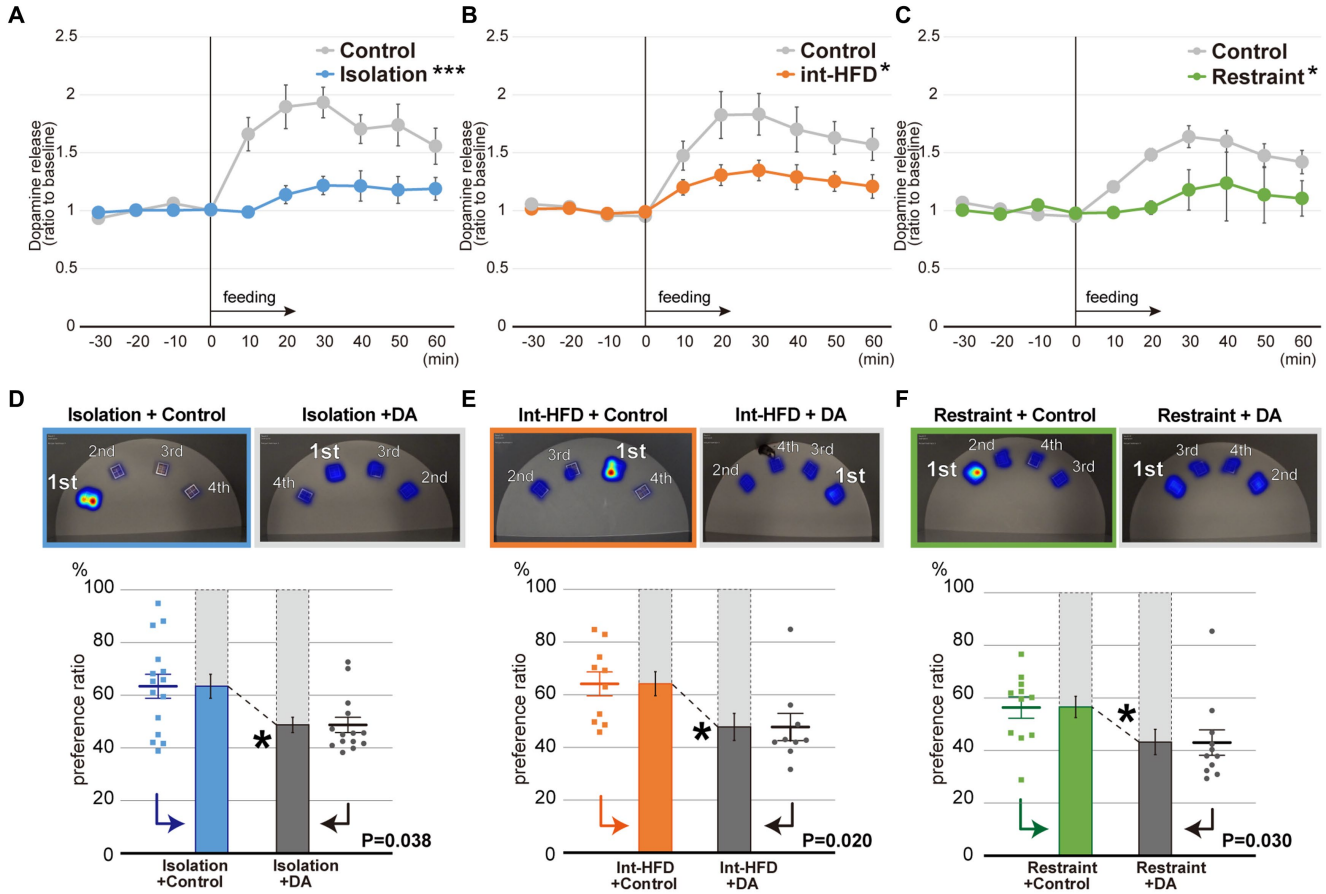
Dopamine levels in the NAcc shell are impaired by psychosocial and physical stresses, whereas dopamine supplementation normalizes the stress-induced fixated feeding. **(A)** *In vivo* microdialysis analysis of mice from the isolation group. Feeding increased the amount of dopamine (DA) in the NAcc shell. The extracellular DA levels were determined by *in vivo* microdialysis and HPLC-ECD. Basal fractions were collected prior to the initiation of feeding at time point 0 (*n* = 8 each, ***significantly different from control group by ANOVA with repeated measures). **(B)** *In vivo* microdialysis analysis of mice from the intermittent HFD group (*n* = 8 for Int-HFD, *n* = 9 for Control, *significantly different from control group by ANOVA with repeated measures). **(C)** *In vivo* microdialysis analysis of mice from the restrained group (*n* = 8 for Restraint, *n* = 9 for Control, *significantly different from control group by ANOVA with repeated measures). **(D)** The spatial feeding patterns of mice in the Isolation + Control groups and Isolation + DA groups. Representative heatmap images are as in Figure 1E. The duration of time spent per bait position was quantified with a motion capture system and the sources ranked as in Figure 1E. Similarly, the amount consumed for each source was ranked in decreasing order (Supplementary Figures S2B–D). Statistical analysis was performed on the primary preference ratio as in Figures 1E–G (*n* = 15 for Isolation + Control, *n* = 14 for Isolation + DA, Mann–Whitney U test). **(E)** The spatial feeding patterns of mice in the Int-HFD + Control and the Int-HFD + DA groups. Representative heatmap images, graphs, and statistical analysis are as above (*n* = 10 for Int-HFD + Control, *n* = 9 for Int-HFD + DA, Mann–Whitney U test). **(F)** The spatial feeding patterns of mice in the Restraint + Control and Restraint + DA groups. Representative heatmap images, graphs and statistical analysis are as above (*n* = 11 for each, Mann–Whitney U test). *p < 0.05, ***p < 0.001. Data are mean ± SEM.

To investigate the effects of the stressors on the reward system, *in vivo* microdialysis analyses were performed to assess dopamine levels in the NAcc shell after feeding on a standard chow diet under all three stress conditions. At 10–30 min post-feeding in the social isolation study, dopamine levels in control mice had increased over basal levels. This dopamine peak was not observed in the socially isolated mice and the degree of dopamine increase was significantly lower in the experimental group compared to the control group after feeding (Figure 2A). Post-feeding dopamine increases in the intermittent HFD and restraint mice were likewise significantly reduced compared to controls (Figures 2B,C). These results indicate that stress can inhibit the post-feeding responsiveness of dopamine in the NAcc shell.

To confirm the relationship between aberrant dopamine levels in the NAcc shell and the impaired feeding behavior patterns, we selectively administered dopamine into the NAcc shell of mice under all three experimental conditions and monitored their feeding behaviors (Supplementary Figure S2A). Dopamine administration reversed fixation on the preferred bait position that had been observed in all three conditions, without affecting overall food consumption or the distance traveled by the mice (Figures 2D–F, Supplementary Figures S2B–D, S3A–L).

### 3.3 The three psychosocial and physical stressors had inconsistent effects on total diet consumed and metabolic states

**Figure 3:**
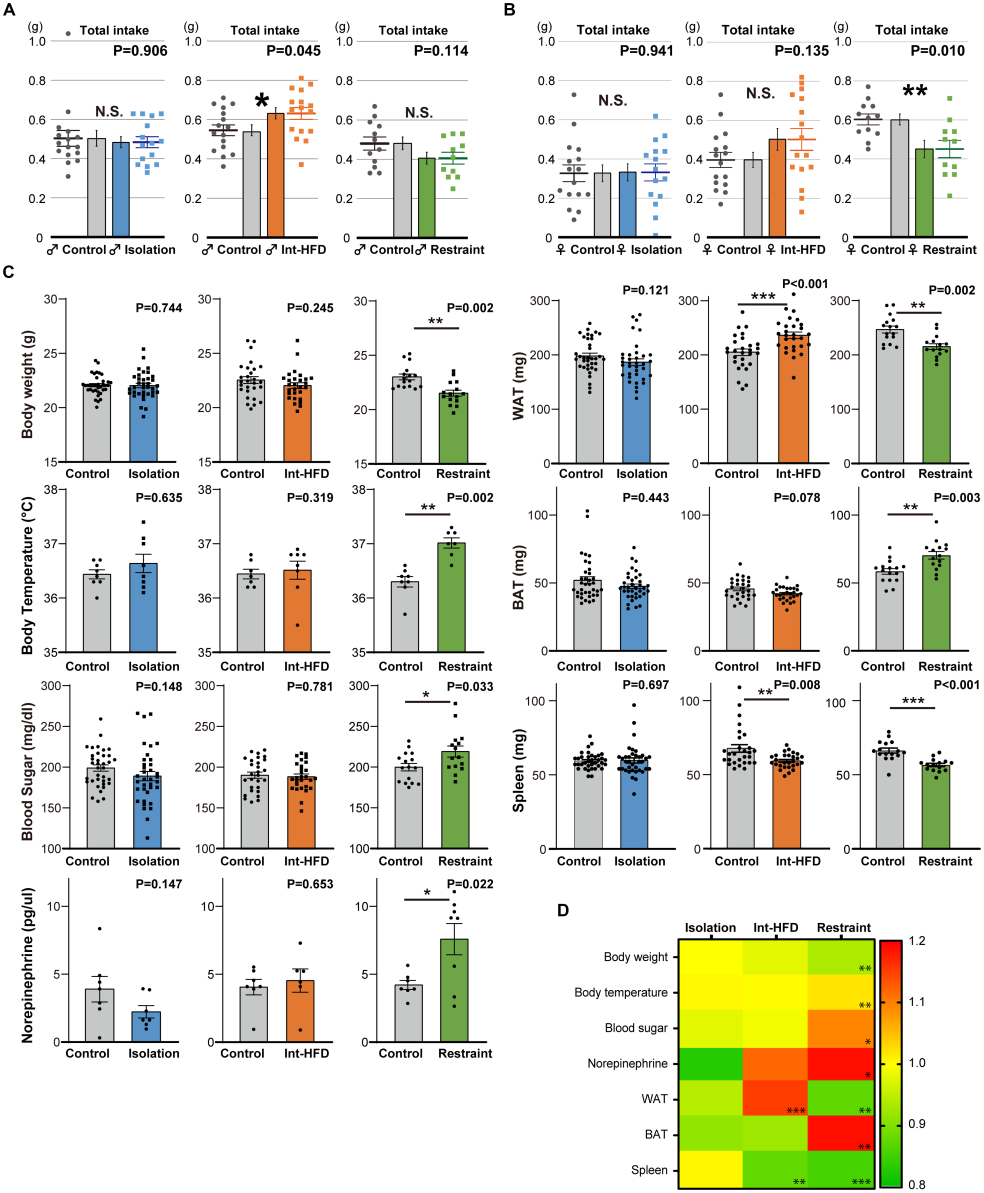
The amount of total consumption and general metabolic factors vary across the three stressor conditions. **(A)** Total bait amount consumed (total intake) by male mice in the three stressor conditions during the real-time monitoring period depicted in Figures 1E–G (*n* = 15 for Isolation, *n* = 16 for Control; *n* = 16 for both Int-HFD and Control; *n* = 10 for Restraint, *n* = 11 for Control, Welch’s *t* test). **(B)** The total amount of bait consumed by female mice across the three experimental stressor conditions throughout the real-time monitoring period in Figures 1H–J (left; *n* = 15 for Isolation, *n* = 16 for Control, middle; *n* = 16 for both Int-HFD and Control, right; *n* = 11 for Restraint, *n* = 12 for Control, Welch’s *t* test). **(C)** Effects of the three stressor conditions (social isolation, Int-HFD, and physical restraint) were assessed in terms of various metabolic factors including, body weight and temperature (rectal temperature), blood sugar, plasma norepinephrine concentration, brown (BAT) and white adipose tissue (WAT) levels, and spleen weight. Results of the measurements across the experimental conditions are depicted via a heatmap (ratio to controls). Note that the norepinephrine data (isolation and restrained) are off scale. **(D)** Quantitative data are shown as comparisons between each respective stress condition (Isolation, blue; Int-HFD, orange; Restraint, green) and controls (Isolation group body temperature—*n* = 8 for both; Int-HFD group body temperature—*n* = 8 for Int-HFD, *n* = 7 for Control; Restrained body temperature—*n* = 7 for Restraint, *n* = 8 for Control; Mann–Whitney U test. Isolation group plasma norepinephrine—*n* = 7 for both; intermittent HFD group plasma norepinephrine—*n* = 6 for Int-HFD, *n* = 7 for Control; Restrained plasma norepinephrine—*n* = 8 for Restraint, *n* = 7 for Control; Welch’s *t* test. Isolation group others—*n* = 36 for both; intermittent HFD group others—*n* = 28 for both; Restrained group others—*n* = 15 for Restraint, *n* = 16 for Control; Mann–Whitney U test). *p < 0.05, **p < 0.01, ***p < 0.001, N.S. denotes not significant. Data are mean ± SEM.

Total diet consumption during the monitored assay period differed across the three stress states. In the intermittent HFD group, the total intake amount was significantly increased in male mice with a similar trend observed in female mice, whereas the opposite was seen in both male and female mice from the physical restraint group (Figures 3A,B). Next, we investigated the effects of the three stressors on metabolic factors: body weight and temperature, blood sugar, plasma concentration of norepinephrine, brown (BAT) and white adipose tissue (WAT) levels, and spleen weight. We found that three experimental states had variable effects (Figures 3C,D). Gene expression in the hypothalamus, BAT, and WAT varied significantly among different stressors. Specifically, mice subjected to social isolation displayed distinct gene expression patterns compared to those in the physical restraint and intermittent HFD groups (Supplementary Figures S4A–D). Although no consistent effects were apparent across the three experimental states in terms of amount of diet consumed and metabolic factors, all of the stressors promoted feeding patterns characterized by a state of fixated feeding.

### 3.4 Selective inhibition of the neuronal circuit from the VTA to the NAcc replicates fixated feeding patterns

**Figure 4:**
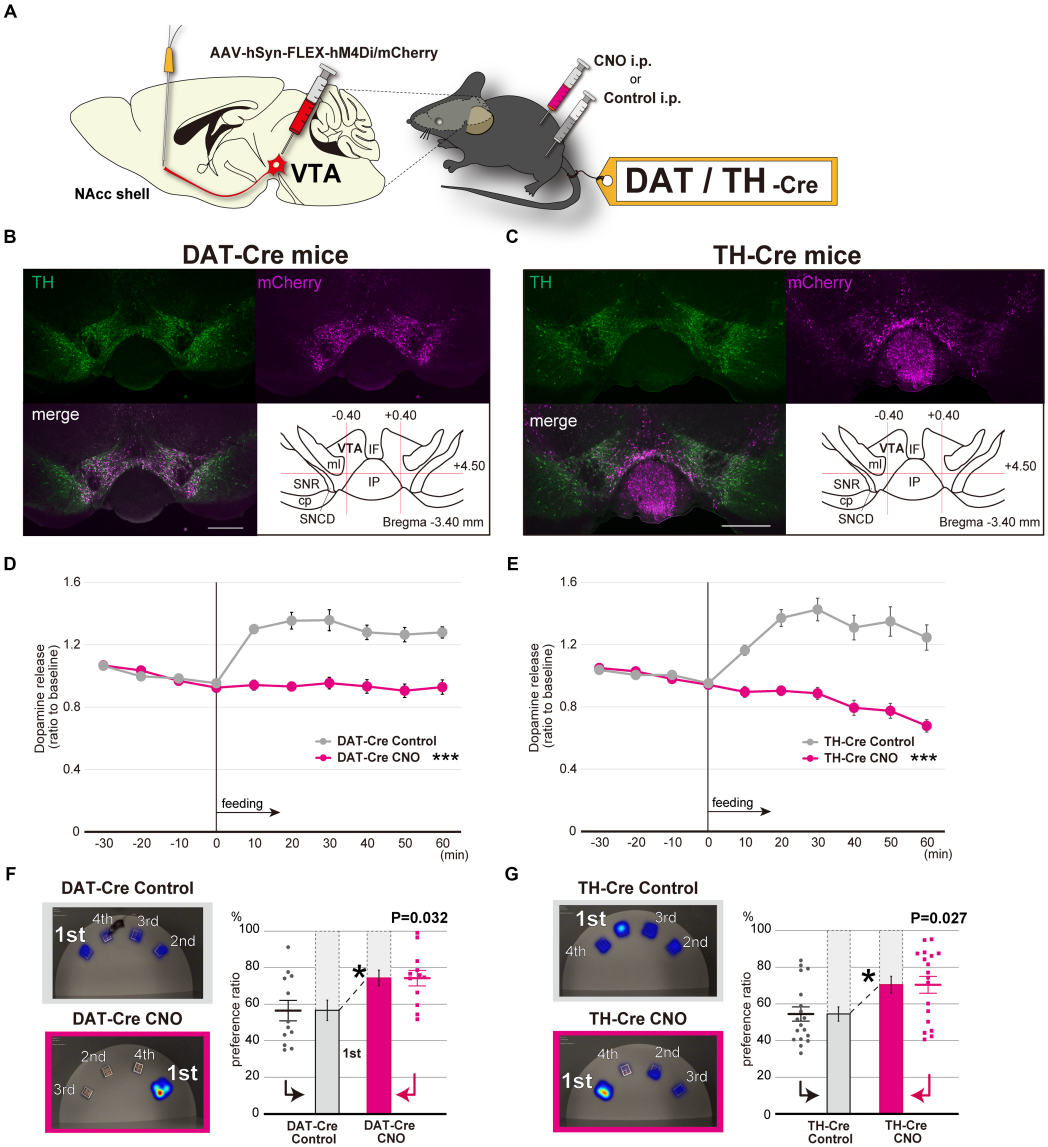
Selective inhibition of the dopaminergic neuronal circuit from the VTA to the NAcc replicates fixated feeding behavior patterns. **(A)** DREADD-based experimental scheme for inhibiting the dopaminergic neurons at the VTA. AAV-hSyn-FLEX-hM4Di/mCherry was injected into the VTA of 6-week-old DAT-Cre or TH-Cre mice (see also Supplementary Figure S5A). **(B)** Immunofluorescent images of dopaminergic neurons in the VTA with mCherry expression in DAT-Cre mice. Sections including the VTA were stained with anti-TH antibodies. Abbreviations used in the Figure: cp, cerebral peduncle; IF, interfascicular nucleus; IP, interpeduncular nucleus; ml, medial lemniscus; SNCD, substantia nigra, compact part, dorsal tier; SNR, substantia nigra, reticular part. Scale bar, 500 μm. **(C)** Immunofluorescent images of dopaminergic neurons in the VTA with mCherry expression in TH-Cre mice. **(D)** *In vivo* microdialysis analysis of DAT-Cre in which AAV-hSyn-FLEX-hM4Di/mCherry was injected into the VTA. Feeding after overnight starvation induced the release of DA in the NAcc shell. Mice were intraperitoneally administered CNO or Control 30 min prior to feeding. The extracellular DA levels of mice were determined as in Figures 2A–C (*n* = 8, ***significantly different from control groups by ANOVA with repeated measures). **(E)** *In vivo* microdialysis analysis of TH-Cre in which AAV-hSyn-FLEX-hM4Di/mCherry was injected into the VTA (*n* = 11, ***significantly different from control groups by ANOVA with repeated measures). **(F)** The spatial feeding patterns of DAT-Cre mice treated with CNO or Control following AAV-hSyn-FLEX-hM4Di/mCherry injection. Representative heatmap images and preference ratios were determined as in Figure 1E (see also Supplementary Figure S5B). Statistical analysis was performed on the most frequently consumed bait ratio (*n* = 12, Wilcoxon signed-rank test). **(G)** The spatial feeding patterns of TH-Cre mice treated with CNO or Control following AAV-hSyn-FLEX-hM4Di/mCherry injection (*n* = 18, Wilcoxon signed-rank test). Representative heatmap images and preference ratios were determined as in Figure 1E (see also Supplementary Figure S5C). *p < 0.05, ***p < 0.001. Data are mean ± SEM.

The dopaminergic neurotransmission circuit from the ventral tegmental area (VTA) to the NAcc shell is largely associated with the reward/motivation system (Arias-Carrión et al., 2010). To investigate the relationship between this dopaminergic circuit and the aberrant feeding behavior patterns, we used the Designer Receptors Exclusively Activated by Designer Drugs (DREADD) system to selectively modulate the dopaminergic neurons at the VTA by incorporating AAVs expressing hM4Di with the FLEX conditional gene expression system in dopamine transporter-Cre transgenic (DAT-Cre) mice and tyrosine hydroxylase-Cre (TH-Cre) transgenic mice (Figure 4A, Supplementary Figure S5A). After selectively inhibiting dopaminergic neuron activity at the VTA in both mice lines (Figures 4B,C), we assessed dopamine levels at the NAcc shell. In response to feeding, dopamine levels increased when administered control solution, but were negligible when they were given clozapine N-oxide (CNO) (Figures 4D,E). In addition, inhibiting dopaminergic neuron activity from the VTA to NAcc shell resulted in fixated feeding in both the DAT-Cre and TH-Cre mice, which indicates that the mesolimbic dopamine-mediated reward system is directly associated with aberrant feeding behavior patterns characterized by fixated feeding (Figures 4F,G, Supplementary Figures S5B,C). To exclude non-specific effects of CNO on the spatial feeding behavior patterns, dopamine levels at the NAcc shell and the spatial feeding patterns were also examined in mice treated with CNO and the control solution. Neither the dopamine levels (Supplementary Figure S6A) nor the feeding patterns (Supplementary Figures S6B,C) were altered by CNO administration.

## 4 Discussion

We found that three different external stressors promoted feeding behavior deviations in food selection (fixated feeding) but had inconsistent effects on either the total amount of diet consumed or a number of metabolic factors. These findings demonstrate that variations in spatial feeding patterns can be used in lieu of total diet consumption as quantifiable indicators of feeding behavior and that they can enable the detection of stressor-mediated and neuropsychiatric disease-associated changes in the reward system.

Intermittent HFD treatment is frequently used as a model for binge eating, which can be caused by various psychosocial stresses (Novelle and Dieguez, 2018). In this study, we sought to detect early traces of feeding behavior alterations that manifest following a relatively short external stimuli application period. Accordingly, we found that mice in the intermittent HFD group exhibited aberrant feeding patterns but maintained body weight despite clear diet consumption per session (Figures 3A–D) (Rospond et al., 2015). Conversely, while mice in the physical restraint stress group also showed similar feeding patterns, they also exhibited significant decreases in body weight with a tendency toward reduced consumption (Figures 3A–D). As stressors can induce both weight gain and loss in humans, the three psychosocial and physical stressors examined here had inconsistent effects on both the amount of bait consumed and body weight. They, however, did consistently induce fixated feedings, indicating that alterations in feeding behavior patterns are inevitable and precede changes in body weight under different stress conditions. Moreover, gene expression profiles of the three stressors were variable. Among them, mice subjected to social isolation displayed distinct gene expression patterns compared to those in the physical restraint and intermittent HFD groups (Supplementary Figures S4A–D). Notably, the CRH and CRHR1 genes, which are known to be upregulated in the pituitary–adrenal (PHA) axis, showed a marked increase in the intermittent HFD and restraint stress groups, indicating a robust stress response in these conditions. This variation could be attributed to the comparatively mild internal stress response in the isolation group, where mice were isolated for only 1 week. Previous research has indicated that significant stress responses are more commonly observed after two or more weeks of exposure (Lin et al., 2018).

It is important to note that across the three experimental conditions-each differing in stress model and characteristics-there were no consistent effects on the amount of diet consumed or on metabolic factors. However, a shared outcome among all stressors was the diminished DA response, leading to alterations in feeding behavior patterns characterized by fixated feeding, regardless of the varying degrees and types of stress applied. Furthermore, administering DA in the NAcc restored normal feeding patterns, but did not significantly alter overall food intake in mice subjected to stress (Supplementary Figures S3A–C). In this study, the absence of a reduction in food consumption, contrary to earlier findings (Boekhoudt et al., 2017), could be attributed to an observed trend of increased movement distance and velocity (Supplementary Figures S3G–L).

This observation underscores the complex interplay between stress, metabolic responses, and feeding behavior, highlighting the role of the dopamine system in mediating the impact of stress on feeding patterns.

We determined that dopamine neurons from the VTA to the NAcc shell participate in the generation of the spatial feeding behavior patterns. Food intake rapidly evokes dopaminergic neuron activity in the VTA, which leads to dopamine release in the NAcc (Gunaydin et al., 2014). A previous study revealed that mice under stress exhibited similar impaired dopamine responses following ethanol intake (Ostroumov et al., 2016). While activation of dopaminergic neurons in the VTA has been reported to suppress reward-driven food consumption (Adamantidis et al., 2011; Mikhailova et al., 2016), our results indicate that feeding behavior patterns are positively regulated by the dopaminergic system from the VTA to the NAcc shell regardless of the amount of food consumed.

Considering that dopamine neurons in the VTA are the center of the reward system and that stresses impact dopaminergic neuronal activity in the mesolimbic dopamine system (Bloomfield et al., 2019), changes in feeding behavior patterns may be indicative of impairments to the reward system that are induced by external stress.

Stress-induced alterations may also reflect innate escape responses in animals since integrated eating behaviors can be beneficial under potential environmental dangers such as predation. From this point of view, external stress-induced changes in the reward system might be an adaptative response by the brain, like that seen in alterations within the hypothalamus or amygdala (Dietrich et al., 2015; Campos et al., 2018; Xu et al., 2019). Furthermore, feeding behaviors can be impacted by some forms of dementia, in particular FTLD and ASD (Ikeda et al., 2002; Ahmed et al., 2016; Bandini et al., 2017). Moreover, these neuropsychiatric diseases affect the mesolimbic reward pathway (Kloeters et al., 2013; Tye et al., 2013; Supekar et al., 2018). As such, altered feeding behavior patterns may be an early biomarker for a spectrum of neuropsychiatric diseases.

In summary, our study showed that psychosocial and physical stress can rapidly alter feeding behavior patterns via the limbic dopamine system. The detection and quantification of these feeding behavior patterns can be powerful tools for investigating the effects of various stressors.

## Data availability statement

The original contributions presented in the study are included in the article/Supplementary material, further inquiries can be directed to the corresponding authors.

## Ethics statement

The animal study was approved by Nagoya University Animal Experiment Committee and the Shiga University of Medical Science Animal Care and Use Committee. The study was conducted in accordance with the local legislation and institutional requirements.

## Author contributions

YF: Conceptualization, Data curation, Funding acquisition, Investigation, Methodology, Resources, Validation, Visualization, Writing – original draft. KaK: Investigation, Methodology, Resources, Validation, Visualization, Writing – original draft, Formal analysis. KE: Investigation, Methodology, Writing – original draft. MI: Investigation, Methodology, Writing – original draft. NI: Investigation, Writing – original draft. DT: Investigation, Writing – original draft. KoK: Methodology, Resources, Writing – original draft. TY: Methodology, Resources, Writing – original draft. AY: Methodology, Resources, Writing – original draft. MK: Methodology, Writing – original draft. HW: Formal analysis, Supervision, Writing – original draft. GS: Supervision, Conceptualization, Funding acquisition, Project administration, Visualization, Writing – original draft, Writing – review & editing. SI: Conceptualization, Funding acquisition, Project administration, Supervision, Visualization, Writing – review & editing, Data curation, Formal analysis, Investigation, Methodology, Resources, Software, Validation, Writing – original draft.
