# Supplementary material for: Stress-impaired reward pathway promotes distinct feeding behavior patterns
Source: Front Neurosci. 2024 May 9;18:1349366. doi: 10.3389/fnins.2024.1349366 (PMC11111882; doi:10.3389/fnins.2024.1349366)
Supplement: Supplementary file 1 [file Data_Sheet_1.pdf]

Supplementary Material

1 Supplementary Figures

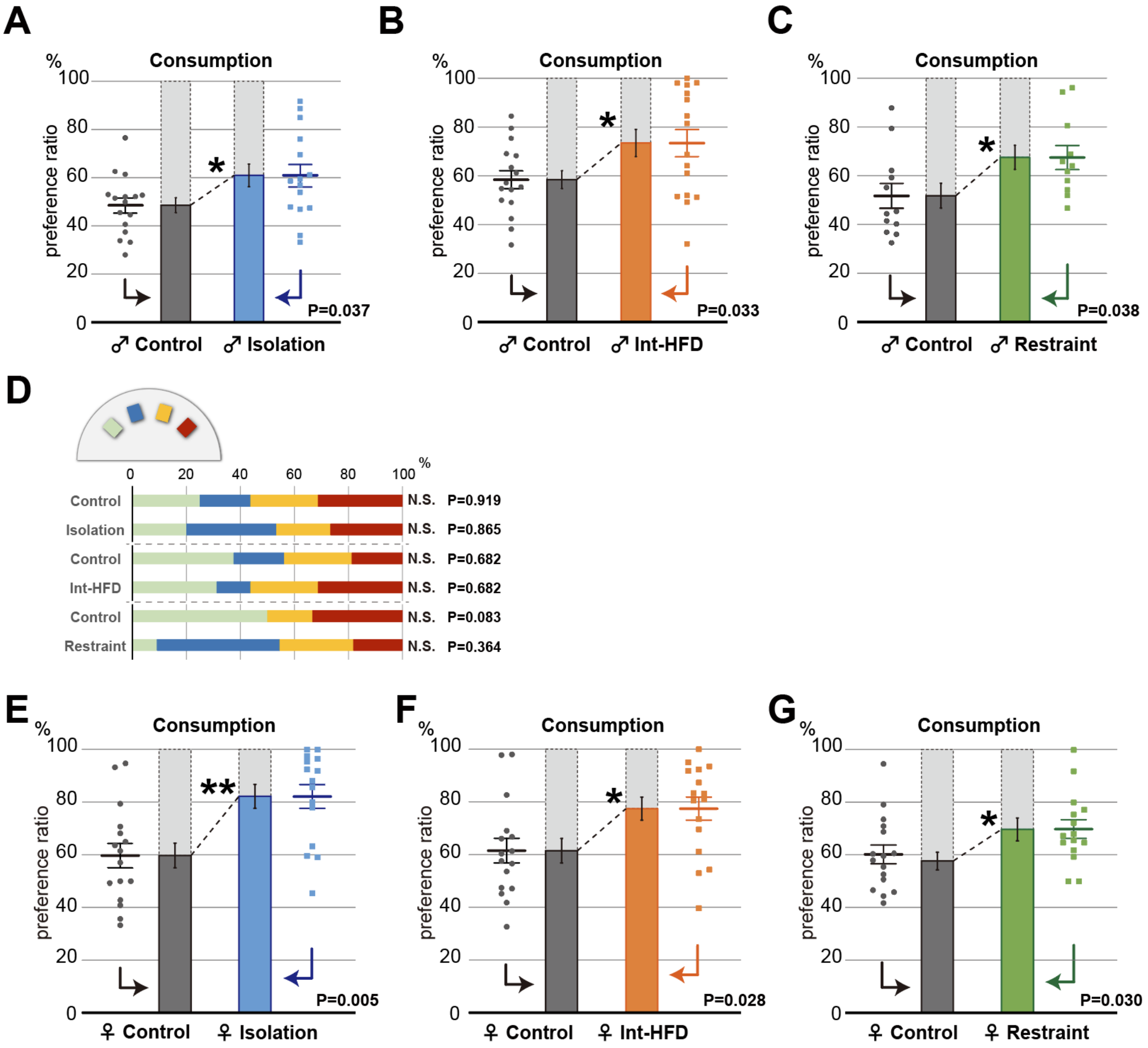

Supplementary Figure S1: Aberrant feeding behavior patterns characterized by fixated feeding were assessed in terms of bait consumption. (**A**) The experiments were the same as those in Figures 1E-G in which the duration at each bait source was quantified by the motion capture system. The spatial feeding patterns of male mice reared in social isolation or group housing (*n* = 15 for Isolation, *n* = 16 for Control, Welch's t test). (**B**) Similar to (A), the spatial feeding patterns of male mice reared on the intermittent HFD or a normal diet (*n* = 16 for both, Welch's t test). (**C**) The spatial feeding patterns of physically restrained and non-restrained male mice (*n* = 11 for Restraint, *n* = 12 for Control, Welch's t test). For Figures 1E-G, the amount consumed from each source was quantified with the sources ranked

in decreasing order of bait consumption. A preference ratio was determined, and statistical analysis was performed on the most frequently consumed bait ratio. (**D**) Bars indicate which bait container was most preferred by the mice in each group. No specific preference was shared across the different groups (Pearson's chi-square test). (**E**-**G**) The amount consumed from each source was quantified with the sources ranked in decreasing order of bait consumption. The experimental setups are the same as those in Figures 1H-J in which the duration of time at each bait source was quantified by the motion capture system. In all cases, the amount consumed from each source was quantified with the sources ranked in decreasing order of bait consumption. The spatial feeding patterns of female mice reared in social isolation or group housing (E; $n = 15$ for Isolation, $n = 16$ for Control, Mann-Whitney U test). The spatial feeding patterns of female mice reared on the intermittent HFD or a normal diet (F; $n = 16$ for both, Welch's t test). The spatial feeding patterns of physically restrained and non-restrained female mice (G; $n = 11$ for Restraint, $n = 12$ for Control; Mann-Whitney U test). $^{*}p < 0.05$, $^{**}p < 0.01$. N.S. denotes not significant. Data are mean ± SEM.

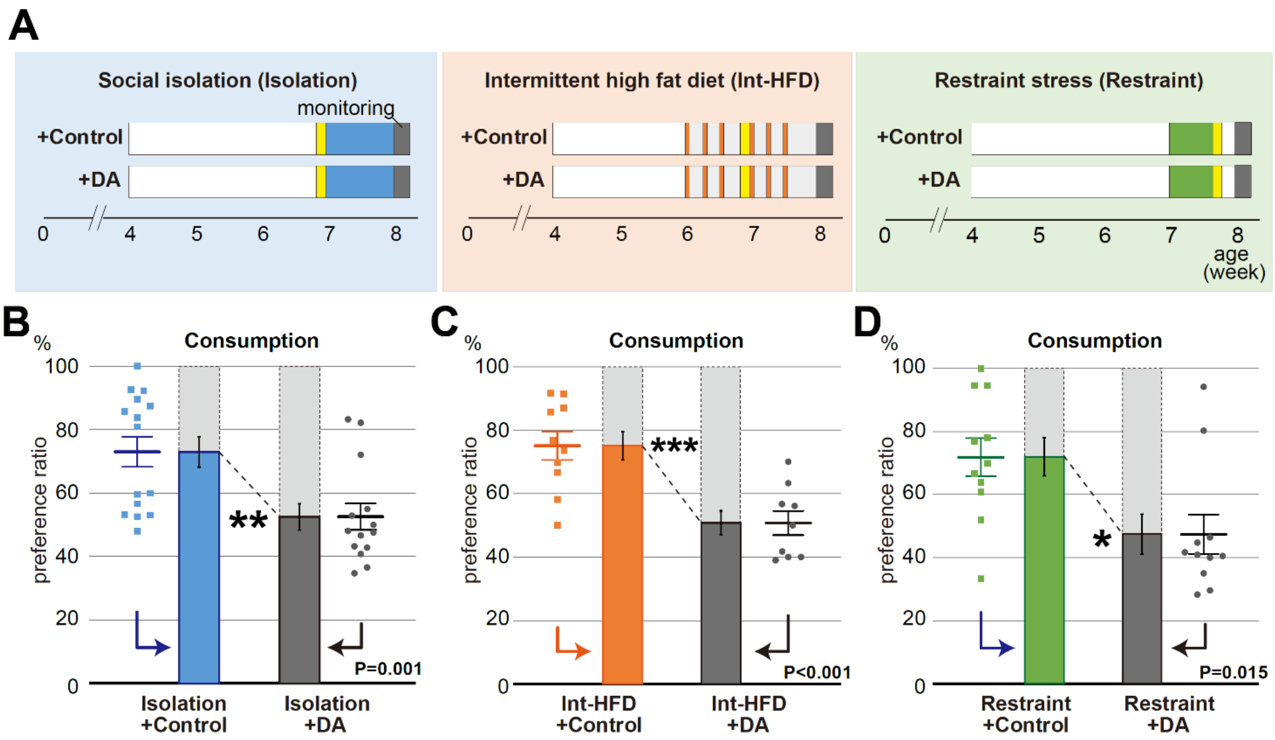

Supplementary Figure S2: Dopamine supplementation in the NAcc shell normalized fixated feeding. (**A**) Schematic of the study paradigm consisting of Isolation + DA and Isolation + Control groups, Int-HFD + DA and Int-HFD + Control groups, and Restraint + DA and Restraint + Control groups. Mice were assigned into two groups, stressors with DA and stressors with 0.5% ASA/Ringer's solution (Control). After stress treatment, the real-time feeding behavior with DA or Control administration was monitored for each mouse after 4 h starvation. Mice were stressed as shown in Figures 1A-C. Mice in the isolation group were housed alone in cages for a week (blue). Mice in the intermittent HFD group were provided access to HFD for 2 h during the day every other day for two weeks (orange). For the restrained group, mice were immobilized with restrainers for 2 h for five consecutive days (green). Yellow boxes indicate the day when cannulas were placed into the NAcc shell. (**B**) The experimental setup is the same as Figure 2D in which the duration of time at each bait source was quantified with the motion capture system. The spatial feeding patterns of mice in the Isolation + DA and Isolation + Control groups were investigated in terms of bait consumption (n = 15 for Isolation + Control, n = 14 for Isolation + DA, Mann-Whitney U test). (**C**) DA effects on the spatial feeding patterns of mice in the Int-HFD were similarly measured in terms of bait consumption. Mice in the Int-HFD + DA and Int-HFD + Control groups (n = 10 for Int-HFD + Control, n = 9 for Int-HFD + DA, Mann-Whitney U test). (**D**) The effects of DA on the spatial feeding patterns of mice in the restrained group were measured in terms of bait consumption. Mice in the Restraint + DA and Restraint + Control groups (n = 11 for both, Mann-Whitney U test). For Supplementary Figures S2B-D, the amount consumed from each source was quantified with the sources ranked in decreasing order of bait consumption. A preference ratio was determined, and statistical analysis was performed on the most frequently consumed bait ratio. *$p < 0.05$, **$p < 0.01$, ***$p < 0.001$. Data are mean ± SEM.

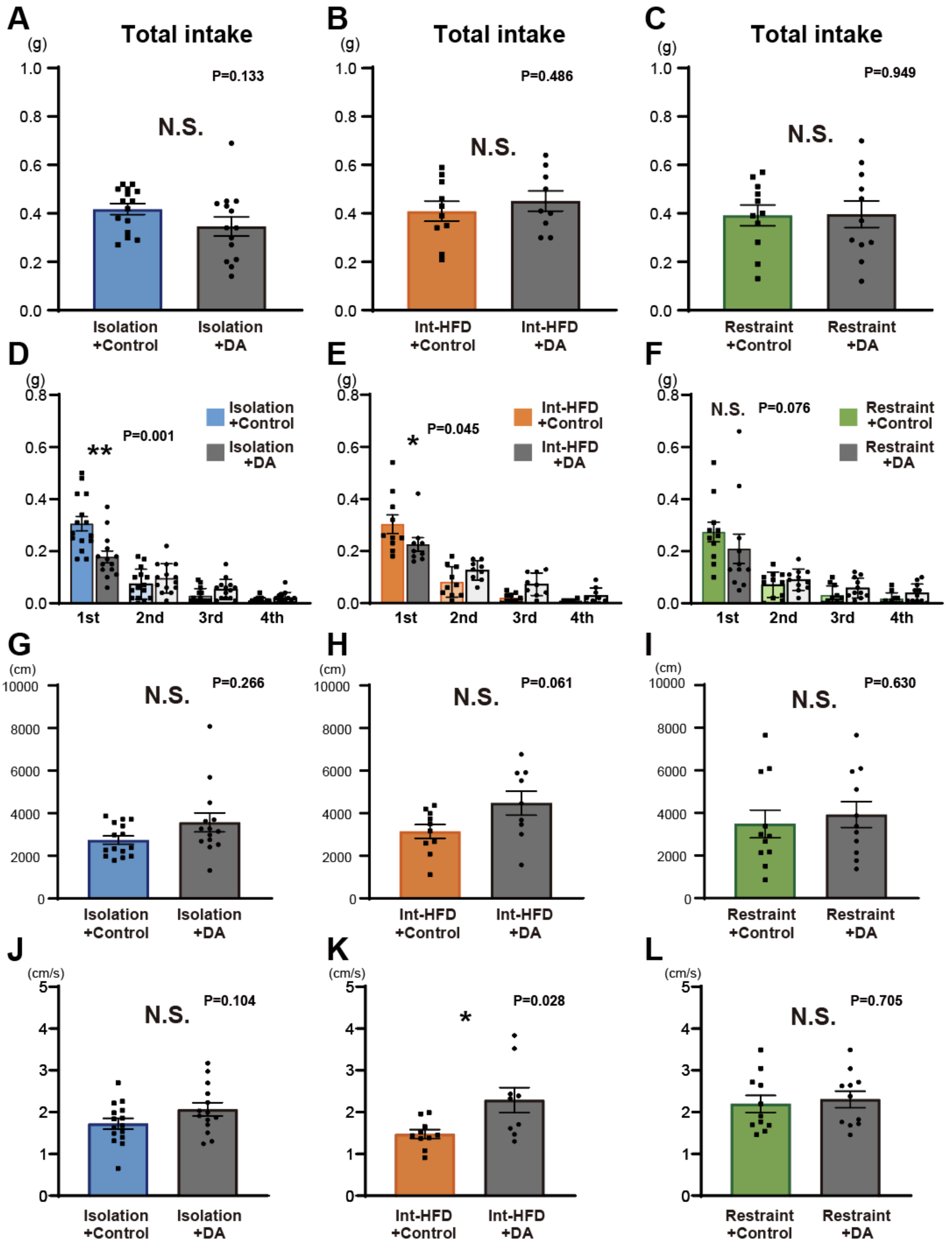
A
Total intake
(g)
P=0.133
N.S.
Isolation +Control
Isolation +DA
B
Total intake
P=0.486
N.S.
Int-HFD +Control
Int-HFD +DA
C
Total intake
P=0.949
N.S.
Restraint +Control
Restraint +DA
D
P=0.001
**
1st
2nd
3rd
4th
E
P=0.045
*
F
N.S.
P=0.076
G
(cm)
P=0.266
N.S.
H
P=0.061
N.S.
I
P=0.630
N.S.
J
(cm/s)
P=0.104
N.S.
K
P=0.028
*
L
P=0.705
N.S.

Supplementary Figure S3: Dopamine supplementation in the NAcc shell didn't show significant changes in total intake during the session. (A-C) Panels detail the total bait consumption across three stressor conditions, both with and without DA supplementation. (A; n = 15 for Isolation + Control, n = 14 for Isolation + DA, Welch's t test, B; n = 10 for Int-HFD + Control, n = 9 for Int-HFD + DA, Welch's t test, C; n = 11 for both, Welch's t test) **(D-F)** The absolute amount of food intake for each rank is illustrated. Statistical analysis was performed on the most frequently consumed bait ratio. (D; n = 15 for Isolation + Control, n = 14 for Isolation + DA, Welch's t test, E; n = 10 for Int-HFD + Control, n = 9 for Int-HFD + DA, Mann-Whitney U test, F; n = 11 for both, Mann-Whitney U test) **(G-I)** The overall distance moved is shown. (G; n = 15 for Isolation + Control, n = 14 for Isolation + DA, Mann-Whitney U test, H; n = 10 for Int-HFD + Control, n = 9 for Int-HFD + DA, Welch's t test, I; n = 11 for both, Welch's t test) **(J-L)** The movement velocity is shown. (J; n = 15 for Isolation + Control, n = 14 for Isolation + DA, Welch's t test, K; n = 10 for Int-HFD + Control, n = 9 for Int-HFD + DA, Welch's t test, L; n = 11 for both, Welch's t test) $*p < 0.05$, $**p < 0.01$, N.S. denotes not significant. Data are mean ± SEM.

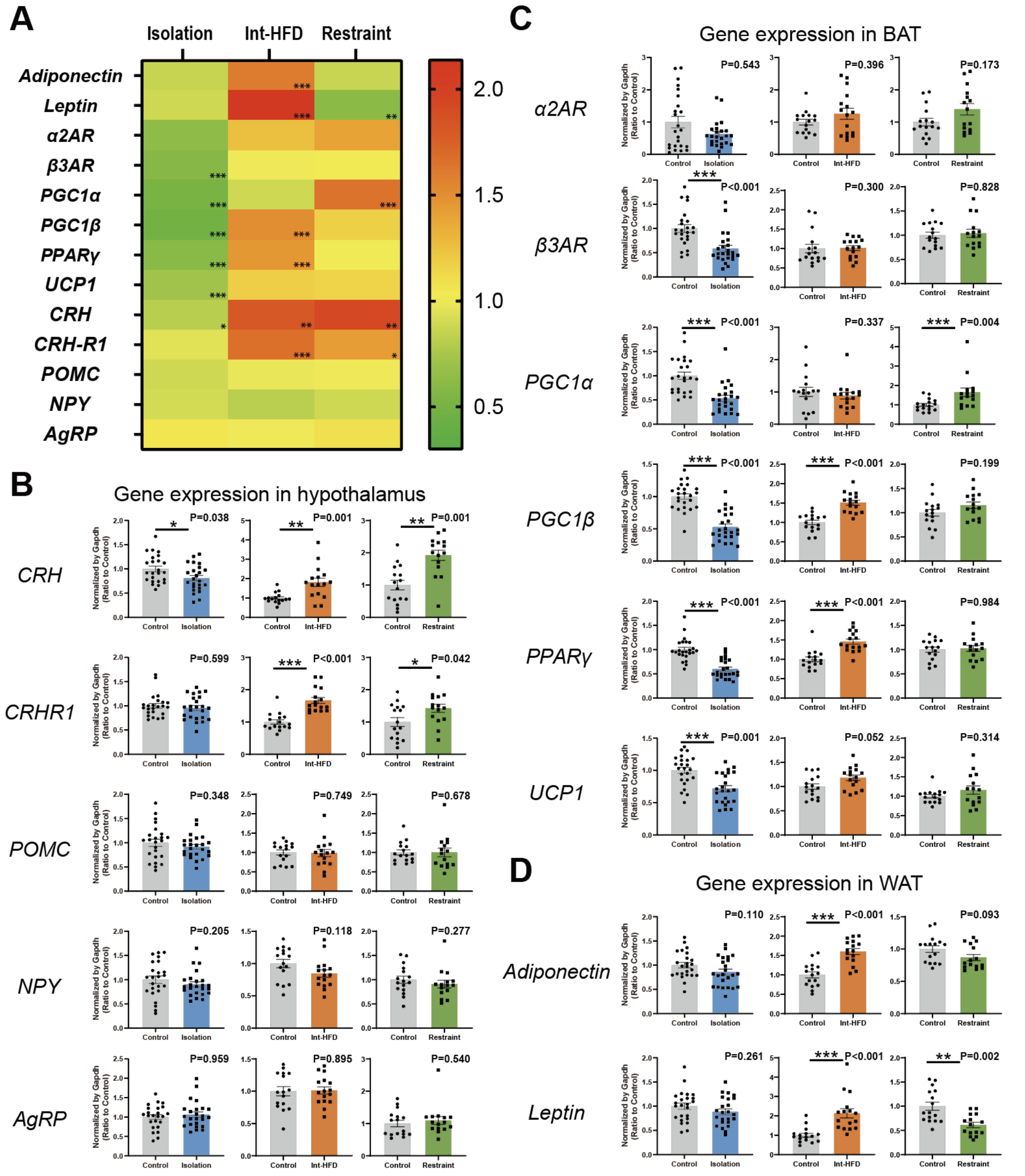

Supplementary Figure S4: Alterations in the hypothalamus, BAT, and WAT-associated gene expression in the three stressor models. (**A**) Alterations of gene expression in the hypothalamus, BAT, and WAT of mice from the three stress conditions (social isolation, Int-HFD, and physical restraint). The expression of *Adiponectin*, *Leptin*, *α2AR*, *β3AR*, *PGC1α*, *PGC1β*, *PPARγ*, *UCP1*, *CRH*, *CRH-R1*, *POMC*, *NPY*, and *AgRP* was measured by qPCR. The relative amount for each transcript was normalized to the housekeeping *Gapdh*. The heatmap summarizes the combined data. (**B**) qPCR-based

analyses of *CRH*, *CRH-R1*, *POMC*, *NPY*, and *AgRP* gene expression in the hypothalamus. Quantitative data are shown as a comparison between the experimental stressor (Isolation, blue; Int-HFD, orange; Restraint, green) and controls (Isolation group – $n = 24$ for both; Int-HFD group – $n = 16$ for both; Restrained group – $n = 15$ for restrained mice, $n = 16$ for control mice; Mann-Whitney U test). (**C, D**) Expression levels of genes in BAT (C: *α2AR*, *β3AR*, *PGC1α*, *PGC1β*, *PPARγ*, and *UCP1*) and WAT (D: *Adiponectin* and *Leptin*). Quantitative data are shown as a comparison between the experimental stressor (Isolation, blue; Int-HFD, orange; Restraint, green) and controls (Isolation group – $n = 24$ for both; Intermittent HFD group – $n = 16$ for both; Restrained group – $n = 15$ for restrained, $n = 16$ for control; Mann-Whitney U test). $*p < 0.05$, $**p < 0.01$, $***p < 0.001$. Data are mean ± SEM.

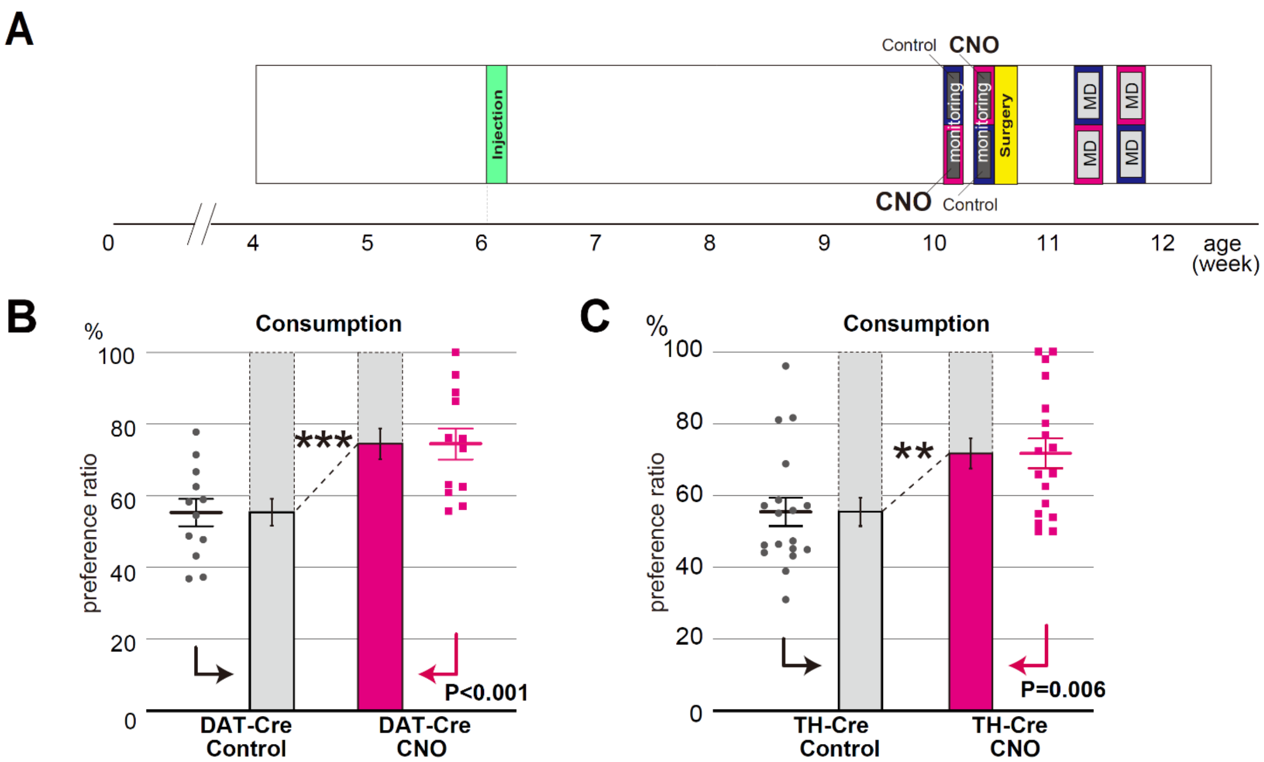

Supplementary Figure S5: Fixated spatial feeding behaviors are replicated by selectively inhibiting the dopaminergic neurons from the VTA to the NAcc shell. (**A**) Schematic of the experimental design used to inhibit the dopaminergic neuronal circuit via the DREADD system. AAV-hSyn-FLEX-hM4Di/mCherry was injected into the VTA of 6-week-old DAT-Cre or TH-Cre mice (green boxes). The real-time feeding behavior of 10-week-old mice was monitored after 4 h starvation. CNO or control was intraperitoneally administered to inhibit the activity of hM4Di positive dopaminergic neurons in the VTA 30 min prior to behavioral monitoring. Dopamine release was measured by *in vivo* microdialysis when mice were 11-weeks-old. Yellow boxes indicate the day when the cannulas were placed into the NAcc shell of mice for the microdialysis experiments. (**B-C**) The spatial feeding patterns of DAT-Cre mice (B) or TH-Cre mice (C) treated with CNO or Control following AAV-hSyn-FLEX-hM4Di/mCherry injection. The amount consumed from each source was quantified with the sources ranked in decreasing order of bait consumption. The experimental setup is the same as Figures 4F, G in which the duration of time spent at each bait source was quantified with a motion capture system. A preference ratio was determined, and statistical analysis was performed on the most frequently consumed bait ratio (B; $n = 12$ for each in DAT-Cre mice, paired t-test, C; $n = 18$ for each in TH-Cre mice; Wilcoxon signed-rank test). $^{**}p < 0.01$, $^{***}p < 0.001$. Data are mean ± SEM.

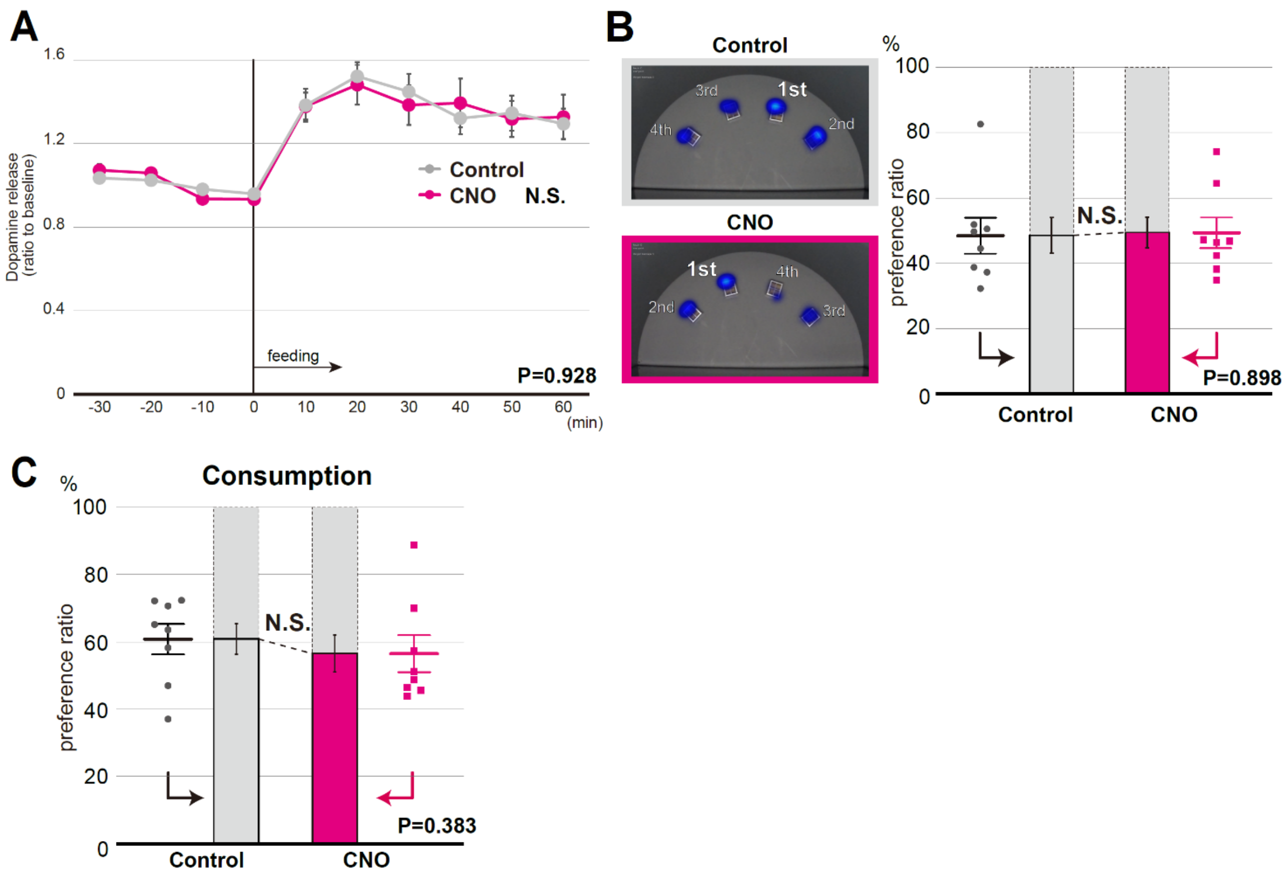

Supplementary Figure S6: CNO administration has no effect on dopamine levels in the NAcc shell or on mouse feeding behavior patterns. To exclude potential nonspecific effects of CNO on either NAcc shell dopamine levels or mouse feeding behavior patterns, mice treated with CNO or Control were similarly examined. (**A**) *In vivo* microdialysis showed no statistical differences between the CNO and Control groups in terms of NAcc shell dopamine levels post-feeding. (n = 12 for each, not significantly different from the control group by ANOVA with repeated measures). (**B-C**) The duration of time spent at each bait position was quantified with a motion capture system (B) and the amount consumed from each source was determined (C). No statistical differences were observed in the preference ratios between the CNO and control groups. Statistical analysis was performed on the most frequently consumed bait ratio (n = 8 for each, B; Wilcoxon signed-rank test, C; paired t-test). There was no statistical difference between the CNO and Control groups. N.S. denotes not significant. Data are mean ± SEM.

2 Supplementary Table

	Forward	Reverse
**white adipose tissue**		
Adiponectin	GCACTGGCAAGTTCTACTGCAA	GTAGGTGAAGAGAACGGCCTTGT
Leptin	GCCAGGCTGCCAGAATTG	CTGCCCCCCAGTTTGATG
**brown adipose tissue**		
α2A-AR	GCTGGTTATTATCGCGGTGT	CAGCGCCCTTCTTCTCTATG
β3-AR	CCTTCCGTCGTCTTCTGTGT	CAGCTTCCTTGCTGGATCTT
PGC-1α	CCCTGCCATTGTTAAGACC	TGCTGCTGTTCCTGTTTTC
PGC-1β	TCCTGTAAAAGCCCGGAGTAT	GCTCTGGTAGGGGCAGTGA
PPAR γ	GTGCCAGTTTCGATCCGTAGA	GGCCAGCATCGTGTAGATGA
UCP1	ACTGCCACACCTCCAGTCATT	CTTTGCCTCACTCAGGATTGG
**hypothalamus**		
CRH	TCTGCGGGAAGTCTTGGAAA	TCCTGTTGCTGTGAGCTTGCT
CRH-R1	TGTTCGGTGAGGGCTGCTA	CGGTCGGTGGAGTACGTGAGT
POMC	CTGCTTCAGACCTCCATAGATGTG	CAGCGAGAGGTCGAGTTTGC
NPY	TCAGACCTCTTAATGAAGGAAAGCA	GAGAACAAGTTTCATTTCCCATCA
AgRP	CAGAAGCTTTGGCGGAGGT	AGGACTCGTGCAGCCTTACAC
Gapdh	TGTCGTGGAGTCTACTGGTG	GGCGGAGATGATGACCCTTT

Supplementary Table S1: Primers used for qPCR in Supplementary Figure S4.

Gene names are shown in italics. Abbreviations used in Supplementary Table S1: AgRP = agouti-related protein; α2AR = α2-adrenergic receptor; β3AR = β3-adrenergic receptor; CRH = corticotropin-releasing hormone; CRH-R1 = corticotropin-releasing hormone receptor 1; NPY = neuropeptide Y; PGC1α = peroxisome proliferator-activated receptor gamma coactivator 1-alpha; PGC1β = peroxisome proliferator-activated receptor gamma coactivator 1-beta; POMC = proopiomelanocortin; PPARγ = peroxisome proliferator-activated receptor gamma; and UCP1 = mitochondrial uncoupling protein 1.
